# The Institutional Worker

**Published:** 1912-01-13

**Authors:** 


THE
### INSTITUTIONAL WORKER
**Being a Special Supplement to "The Hospital"**

### Editor's Notices.

**Contributions:**

Contributions should be written, or preferably typed, on one side of the paper only, and all articles sent in are accepted upon the distinct understanding that they are forwarded to The Hospital only.

The Editor cannot undertake to return MSS. not used, but when a stamped directed envelope is enclosed the MSS. may be returned if a special request is made.

Accepted articles and paragraphs of news will be paid for after publication at the scale rate.

**Address:**

To prevent delay all contributions and letters on editorial business must be addressed exclusively to the Editor, "The Hospital," 29 Southampton Street, Strand, London, W.C. It is important that this regulation shall be strictly observed.

**Correspondence:**

Correspondence on all subjects is invited. The name and address of correspondents must be given as a guarantee of good faith, but not necessarily for publication.

**Special Articles:**

Special articles are invited, and questions, inquiries, and paragraphs upon all matters relating to the work, administration, and management of general, special, mental, fever, cottage and convalescent hospitals, sanatoria, homes, institutions, societies and organisations for the treatment or care of the sick, injured and dependents of all classes. Every matter affecting the interests and welfare of the staff of all grades working in these institutions will receive special consideration.

**Administrative Medicine, Research and Items of News:**
Special terms will be paid for approved articles on subjects in this wide field by experts who have made some section of it their special study and interest. We wish to give prominence to every movement tending to advance accuracy of diagnosis, efficiency in treatment, and the development of modern methods to eradicate disease and promote the welfare of the suffering.

**A Bureau of Information:**
We invite inquiries and applications for information and help in providing for that numerous class of sufferers who require special care or house-room which they cannot obtain in their own homes or secure for themselves. We cannot, however, prescribe or recommend practitioners.

**Books for Review:**
Publishers are particularly requested to send advance proofs of any new books of importance whenever possible, as the Editor has made arrangements to publish immediate reviews on a new plan.

**Photographs, Plans, Blocks, and Illustrations:**
It is requested that wherever possible MSS. may be accompanied by illustrations in any of the above forms. The name of the sender and of the article to which it belongs should be written on the back of each photograph, drawing, or block for purposes of identification.

**Local Papers:**
Newspapers containing reports or news paragraphs should be marked and addressed to the Sub-Editor.

**The Coupon System:**
A coupon will be found at the bottom of the third inside page of the cover each week. This coupon must be attached to every question or inquiry to which an answer is desired in The Hospital.

### Manager's Notices.

Letters relating to the publication, sale, and advertising departments of The Hospital must be addressed to the Manager, "The Hospital," The Hospital Building, 28 and 29 Southampton Street, Strand, London, W.C.

**Subscriptions** which may begin at any time, are payable in advance. Cheques and Post-Office orders, crossed London County and Westminster Bank, Covent Garden branch, should be made payable to the Manager of The Hospital and addressed to him at The Hospital Building, 28 and 29 Southampton Street, Strand, London, W.C.

**Advertisements:**

All advertisements for The Hospital must reach the Manager not later than Wednesday morning in each week. For scale of charges see pages v and 2.

**Classified Advertisements:**

All small advertisements will be classified, for this section of the paper is widely read and of special interest. We wish to encourage those wanting appointments who are attracted to institutional work, and therefore offer them the reduced rate of *twenty-one words and under for* 1s., and *for every ten and part of ten words beyond,* 6*d.*

### POINTS FOR INSTITUTION AUTHORITIES.

The economical management of an Institution depends very greatly upon purchasing supplies in the cheapest market.

It is often impossible locally to secure the highest quality at the lowest prices.

In consequence, a section will be reserved in this part of the paper for announcements of Institutions inviting Tenders for Contracts.

This will enable Authorities of Institutions to secure tenders for supplies from all over the country, and ensure purchases at the lowest possible prices consistent with quality.

The charge for Tenders is 6s. per inch, and for this small outlay many pounds may be saved on supplies of all descriptions.

**Special Rates** will be quoted for those institutions which agree to send all their vacancy, contract, and public notices to The Hospital each year, so as to make it a file of such announcements for ready reference.

### POINTS FOR TRADE ADVERTISERS.

The Hospital is the organ of Institutions, whether of a Voluntary Character or those under Local Government, Poor Law and Municipal Authorities.

It is read by all Executive Officials and those responsible for the Construction, Management, and Equipment of Public Establishments.

It is also the Journal of the worker engaged in the Medical, Health and Preventive services.

There is no medium more appropriate and effective in the promotion of business in these directions than The Hospital.

### POINTS FOR INSTITUTIONAL WORKERS.

A very large number of Appointments Vacant appear every week in the columns of The Hospital.

If you cannot find a position suited to your requirements, make your wishes known through our Situations Wanted Column.

### APPOINTMENTS VACANT.

### Poor-Law Vacancies.

### Appointments Vacant.

ASSISTANT DRILL MASTER (15s.), Central London School District. Mr. G. P. Morrell, Central London District School, Hanwell, W.

ASSISTANT MATRON (£17), Rye Union. Mr. T. J. Smith, Flushing House, Rye. Jan. 19.

ASSISTANT MATRON AND HEAD ATTENDANT (£26), Fulham Board of Guardians. Mr. E. J. Mott, C. to G., 129 Fulham Palace Road, W. Jan. 17.

ASSISTANT NURSE (£25), Gainsborough Union. Mr. D. M. Robbs, C. to G., Union Office, Gainsborough. Jan. 15.

ASSISTANT NURSE (£20), Falmouth Union. Mr. R. N. Rogers, C. to G., Union Offices, Falmouth. Jan. 23.

ASSISTANT NURSE (£25), Glanford Brigg Union. Mr. F. C. Hett, C. to G., 11 Bigby Street, Brigg. Jan. 17.

ASSISTANT NURSE (£25), Windsor Union. P. Lovegrove, C. to G., 3 Park Street, Windsor. Jan. 13.

ASSISTANT NURSE (£22), Bridport Union. J. J. Roper, C. to G., Bridport.

ATTENDANTS (Male and Female) (£30-£20), Doncaster Union. Mr. H. M. Marshall, C. to G., Union Offices, Doncaster. Jan. 15.

BABY ATTENDANT (£30), Burnley Union. Mr. J. S. Horn, C. to G., Union Offices, Burnley. Jan. 17.

BOYS' (MALE) COOK ATTENDANT (£30), Farnham, &c., District School. Superintendent, District Schools, Crondall, Hants.

CERTIFICATED MIDWIFE (£35), St. Marylebone Guardians. Matron, Northumberland Street Workhouse, Baker Street, W.

CHARGE NURSES (£30), Rochford Union. Mr. F. Gregson, C. to G., 46 Alexandra Street, Southend-on-Sea. Jan. 16.

CHIEF MALE ATTENDANT (£65), Cornwall County Asylum. Medical Superintendent, Cornwall County Asylum, Bodmin. Jan. 22.

CHIEF MALE ATTENDANT ON IMBECILES (£35), Southampton Union. Mr. A. J. Walden, C. to G., Southampton. Jan. 13.

CHILDREN'S CARETAKER (Female) (£20), Sculcoates Union. Mr. J. H. Wild, C. to G., Hull. Jan. 18.

CLERK (15s.), Shoreditch Guardians. Mr. R. Clay, 213 Kingsland Road, N.E. Jan. 13.

COOKERY TEACHER, ETC. (£80), St. Pancras Guardians. Mr. J. E. P. Hall, Town Hall, Pancras Road, N.W.

DRILL MISTRESS (£35), Parish of St. Pancras. Mr. J. E. P. Hall, C. to G., Town Hall, Pancras Road, N.W. Jan. 19.

DRILL MISTRESS AND GIRLS' HEAD ATTENDANT (£30), Holborn Union. Matron, Holborn Schools, Mitcham.

FOSTER-MOTHER (£25), Tendring Union. Matron, Tendring Workhouse, Weeley, Essex.

GIRLS' AND INFANTS' ATTENDANT (£25), Chesterfield Union. R. F. Hartwright, C. to G., Union Offices, Chesterfield. Jan. 13.

HEAD NURSE (£45), East Preston Union. Mr. A. Shelley, C. to G., Littlehampton. Jan. 13.

HEAD NURSE (£40), West London School District. F. G. Beeching, clerk to managers, Ashford, Middlesex. Jan. 25.

HEAD NURSE (£35), Glanford Brigg Union. F. C. Hett, C. to G., 11 Bigby Street, Brigg. Jan. 18.

HEAD NURSES (three) (£25), Holborn Union. Matron, The Infirmary, Archway Road, N.

INFANTS' NURSE (£25), Bucklow Union. G. Leigh, C. to G., Union Offices, Knutsford. Jan. 22.

MALE IMBECILE ATTENDANT (£26), Barton-upon-Irwell Union. Mr. J. W. Whitworth, C. to G., Union Offices, Patricroft. Jan. 16.

MALE LUNATIC ATTENDANT (£30), Parish of St. Marylebone. Master, Workhouse, Northumberland Street, W.

MALE NURSE FOR EPILEPTICS, ETC. (£30), Lambeth Parish. Master of the Workhouse, Lambeth.

MASTER AND MATRON (£60-£45), Dorking Union. Mr. W. James, C. to G., Dorking. Jan. 25.

MIDWIFE (certificated) (£35), St. Marylebone Guardians. Matron, Workhouse, Northumberland Street, W.

NIGHT SISTER (£38), Sculcoates Union. J. H. Wild, C. to G., 12 Harley Street, Hull. Jan. 17.

NIGHT SISTER (£35), Stockport Union. Matron, Union Workhouse, Shaw Heath, Stockport.

NURSE (£30), Guisborough Union. Mr. W. Richardson, C. to G., Guisborough. Jan. 16.

NURSE (£30), Wellington (Salop) Union. Mr. R. Gwynne, C. to G., Wellington, Salop. Jan. 18.

NURSE (£30), Falmouth Union. Mr. R. N. Rogers, C. to G., Union Offices, Falmouth. Jan. 23.

NURSE (£35), Tendring Union. Matron, Tendring Workhouse, Weeley, Essex.

NURSE (£30), Elham Union. E. Lovick, C. to G., 29 Bouverie Square, Folkestone. Jan. 17.

NURSE (£35), Tendring Union. Matron, The Infirmary, Tendring, Weeley, S.O.

NURSES (2) (£31 11s.), East Preston Union. Mr. A. Shelley, C. to G., Littlehampton. Jan. 13.

SISTER (£35), Medway Union. A. Reynolds Norman, C. to G., 22 High Street, Chatham. Jan. 16.

STEWARD'S CLERK (15s. weekly), Kensington Guardians The Steward, Kensington Infirmary, Marloes Road, W.

SUPERINTENDENT OF FEMALE LUNATIC WARDS (£55), South Manchester Guardians. D. S. Bloomfield, C. to G., All Saints, Manchester. Jan. 16.

TEMPORARY CHARGE NURSE (£32), Fulham Guardians. Matron, Infirmary, St. Dunstan's Road, W.

TEMPORARY RELIEVING OFFICER (£2), Brighton Parish. Mr. B. Burfield, C. to G., Brighton.

WARD SISTER (£30), Bermondsey Parish. Matron, Bermondsey Infirmary, Lower Road, Rotherhithe, S.E.

WARD SISTER (£35), Wandsworth Union. F. W. Piper, C. to G., Union Offices, St. John's Hill, Wandsworth.

WARD SISTER (£32), Prestwich Union. E. W. Ogden, C. to G., Union Offices. Cheetham Hill Road, Manchester. Jan. 23.

WARD SISTER (£38), Sunderland Union. W. P. Brantingham, C. to G., 17 John Street, Sunderland. Jan. 17.

### Situations Vacant.

ASSISTANT COOK (£22), Bethnal Green Guardians. The Matron, Bethnal Green Infirmary, Cambridge Road, N.E.

ASSISTANT COOK (FEMALE) (£20), Greenwich Union. Mr. S. Saw, C. to G., Greenwich. Jan. 13.

ASSISTANT COOK-GENERAL (£18), Kingston-on-Hull Incorporation. Mr. R. H. Winter, C. to G., St. Mary's Chambers, Hull. Jan. 16.

COOK (£25), Wayland Union. Mr. F. Robinson, C. to G., Watton, Norfolk. Jan. 13.

COOK (£40), Stockport Union. Matron, Union Workhouse, Shaw Heath, Stockport.

COOK AND KITCHENMAID (£20), Holborn Union. Matron, Holborn Schools, Mitcham.

COOK AND MATRON'S ASSISTANT (£25), Wimborne and Cranborne Union. Mr. M. Luff, C. to G., Union Offices, Wimborne. Jan. 26.

GATE PORTER (£30), Parish of St. Pancras. Mr. J. E. P. Hall, C. to G., Town Hall, Pancras Road, N.W.

KITCHENMAID (£20), Edmonton Union. Matron, Infirmary, 77 Bridport Road, Upper Edmonton.

LAUNDRESS (£27 10s.) Thetford Union. Mr. J. Houchen, C. to G., Thetford.

LAUNDRESS (£25), Tendring Union. Matron, Tendring Workhouse, Weeley, Essex.

PORTER (£24), Wimborne and Cranborne Union. Mr. M. Luff, C. to G., Union Offices, Wimborne. Jan. 18.

PORTER (£25), Basingstoke Union. Mr. H. W. Chandler. C. to G., Basingstoke. Jan. 19.

PORTRESS AND RECEIVING WARD ATTENDANT (£25), Chelsea Guardians. Mr. C. W. Shepherd, 250 King's Road, Chelsea, S.W. Jan. 18.

SENIOR HOUSEMAID (£18), Edmonton Union. Matron, Infirmary, 77 Bridport Road, Upper Edmonton.

### Institutional and Administrative Vacancies.

### Appointments Vacant.

LADY SUPERINTENDENT (£65), West Kent General Hospital, Maidstone. Secretary. Jan. 15.

MATRON (£80), Oldham Royal Infirmary. E. Lionel Blake, Superintendent-Secretary. Jan. 15. (See advt.)

NURSE (£27), Royal Hospital, Chelsea. Matron.

NURSE-MATRON (£30), Leominster Cottage Hospital. Mr. J. B. Dowding, Secretary.

THEATRE SISTER (£35), Oldham Royal Infirmary. E. Lionel Blake, Superintendent-Secretary. Jan. 15. (See advt.)

SISTER (£35), Oldham Royal Infirmary. E. Lionel Blake, Superintendent-Secretary. Jan. 15. (See advt.)

### Municipal Vacancies.

### Appointments Vacant.

ASSISTANT NURSE (£30), Erith Urban District Council Isolation Hospital. J. K. Allerton, Clerk, Council Offices, Erith. Jan. 17.

ASSISTANT SANITARY INSPECTOR (£120), Heston and Isleworth U.D.C. Mr. H. J. Baker, Clerk to the Council, Council House, Hounslow. Jan. 25.

ASSISTANT SANITARY INSPECTOR (35s.) Middlesbrough T.C. Mr. P. Kitchen, Town Clerk, Middlesbrough. Jan. 31.

BOROUGH SURVEYOR AND ENGINEER (£600), Southport T.C. Mr. J. E. Jarratt, Town Clerk, Southport. Jan. 15.

CLERK (£120), Glamorgan C.C. Mr. A. W. Fox, County Accountant, 4 Westgate Street, Cardiff. Jan. 13.

CLERK AND ACCOUNTANT (£150), Hebden Bridge U.D.C. Mr. A. R. Edwards, Clerk to the Council, Council Offices, Hebden Bridge. Jan. 17.

HEALTH VISITOR (£90), County Borough of Bournemouth. H. Ashling, Town Clerk, Municipal Offices, Bournemouth. Jan. 22.

INSPECTOR AND ADJUSTOR OF WEIGHTS AND MEASURES (£120), Wakefield T.C. Mr. W. W. Greenhalgh, Town Hall, Wakefield. Jan. 13.

LADY HEALTH VISITOR (£85), Lincoln Corporation. Mr. C. J. Coleman, Medical Officer of Health, Lincoln. Jan. 23.

NURSE (£36), Epsom U.D. Council Isolation Hospital. E. G. Wilson, Clerk, Church Street, Epsom. Jan. 15.

PROBATIONER (£15), Chesterfield Joint Committee Infectious Diseases Hospital. J. Middleton, Law Clerk, Gluman Gate, Chesterfield. Jan. 13.

PROBATIONERS (£18), Willesden U.D. Council Isolation Hospital. Matron.

PROBATIONER (£10), Coventry Isolation Hospitals. Matron, City Hospital, Coventry.

